# Comprehensive Assembly and Comparative Analysis of Chloroplast Genome and Mitogenome of *Prunus salicina* var. *cordata*

**DOI:** 10.3390/genes16060660

**Published:** 2025-05-29

**Authors:** Ruyu Liao, Mengshi Zhao, Qin Lan, Song Peng, Fengqiang Lin, Zhaolong Li

**Affiliations:** 1 Fruit Research Institute, Fujian Academy of Agriculture Science, Fuzhou 350013, China; liaoruyu@126.com (R.L.); lqin.a@foxmail.com (Q.L.); 2 Institute of Animal Husbandry and Veterinary Medicine, Fujian Academy of Agricultural Sciences, Fuzhou 350013, China; 13375001253@163.com (M.Z.); pengsong@faas.cn (S.P.); linfengqiang@faas.cn (F.L.)

**Keywords:** *Prunus salicina* var. *cordata*, chloroplast genomes, mitochondrial genomes, phylogeny analysis

## Abstract

**Background:** *Prunus* plants are widely distributed across Asia and Europe, yet their intricate phylogenetic relationships pose significant challenges for systematic studies and interspecies identification. **Objectives**: To clarify the mitochondrial and chloroplast genomes of *Prunus salicina* var. *cordata*, and to reveal its evolutionary relationship and historical gene flow with domesticated cherries. **Methods**: In this study, we assembled, annotated, and analyzed the first mitochondrial and chloroplast genomes of *P. salicina* var. *cordata*, a species within the *Prunus* genus. **Results**: The mitochondrial genome was found to be 484,858 base pairs in length, exhibiting a typical circular conformation. Phylogenetic analysis revealed a close evolutionary relationship between *P. domestica* and *P. salicina*, suggesting historical gene flow between these two species last genomes; mitochondrial genomes; phylogeny analysis. **Conclusions**: To provide a genomic basis for resolving the phylogenetic controversies within the Li-associated plants, elucidating their evolutionary mechanisms, and formulating breeding strategies.

## 1. Introduction

The genus *Prunus* belongs to the Rosaceae family and includes a wide array of economically significant fruit-bearing species, such as plums, cherries, almonds, peaches, and apricots. This genus is renowned for its diversity, comprising more than 430 species that are predominantly distributed across temperate regions of the Northern Hemisphere [1]. *Prunus* species are vital for horticulture, agriculture, and landscape architecture due to their ornamental and nutritional value [2]. *P. salicina* (Japanese plum) and *P. domestica* (European plum) are two notable species within this genus [3]. *P. salicina*, originally native to China, were widely cultivated in Japan, and later introduced to other parts of the world, including the United States. *P. salicina* var. *cordata*, commonly known as the Heart Cherry, is a delightful fruit tree that belongs to the *Prunus* genus within the Rosaceae family [4]. This distinct variety of *P. salicina*, native to East Asia, has garnered significant attention due to its unique characteristics and its valuable contribution to horticulture and culinary traditions. *P. salicina* is renowned for its strikingly heart-shaped fruit, which is a small-to-medium-sized cherry with a deep-red-to-almost-black skin when ripe. The name “cordata” is derived from the Latin word “cordatus”, which means heart-shaped, aptly describing the distinctive appearance of the fruit. The flesh of *P. salicina* is juicy, sweet, and succulent, making it a sought-after choice for fresh consumption and various culinary applications. In addition to its appealing fruit, *P. salicina* is appreciated for its ornamental qualities. Its tree, with its glossy green leaves, provides an attractive addition to gardens and landscapes, especially during the flowering season when it produces delicate, white blossoms that create a visually pleasing contrast to the dark, cherry-laden branches. However, despite their global importance, critical gaps persist in understanding the organelle genome dynamics of these species, particularly the mitochondrial (mtDNA) and chloroplast (cpDNA) genomes, which are essential for resolving taxonomic ambiguities and elucidating evolutionary relationships.

*P. domestica* is characterized by its round, juicy fruit and smooth skin. *P. domestica* is believed to have originated in the region around the Caucasus and western Asia. It produces a diverse range of plum types, from sweet and juicy dessert plums to drier prunes. Taxonomically, both the species fall under the subgenus Prunophora. *P. domestica* is further classified into several subspecies and cultivars, which vary significantly in terms of fruit size, color, and taste [5]. The complex hybridization history of *P. domestica* makes it a particularly intriguing subject for genetic and genomic studies.

Recent ecological studies on *Prunus* species have focused on their adaptability to various climatic conditions and their role in supporting biodiversity [6]. *P. salicina* and *P. domestica* are known for their resilience and ability to thrive in different environmental settings, which has implications for their cultivation and conservation. Molecular research has advanced our understanding of the genetic diversity within the *Prunus* genus [7]. Genome-wide association studies (GWASs) and high-throughput sequencing technologies have been employed to identify the genes responsible for important agronomic traits, such as disease resistance, fruit quality, and stress tolerance [8]. In particular, studies on the chloroplast and mitochondrial genomes of *Prunus* species have provided insights into their evolutionary history and phylogenetic relationships [9].

Plant organelle genome research has become an important area of study for understanding the evolutionary dynamics and functional aspects of *Prunus* species. Mitochondria are integral cellular components with pivotal roles in the control of energy metabolism, apoptosis, aging, and various diseases. Their double-stranded mitochondrial DNA (mtDNA) serves as a valuable molecular marker for systematic studies, owing to its straightforward structure, rapid evolutionary rate, abundant copies, and ease of isolation [10]. These characteristics render it a convenient and effective resource for investigating genetic relationships and phylogenetic patterns. Mitogenomes hold significant importance in molecular biology research, providing essential insights into evolutionary relationships, population history, and genetic diversity. They are employed for tasks such as species identification, classification, and phylogenetic analysis, revealing the phylogenetic relationships of species and contributing to the reconstruction of a genus’s evolutionary tree. Mitochondrial genomes also facilitate the examination of gene flow, migration patterns, and genetic diversity among species. The mitochondrial genome (mitogenome) of plants is relatively large and complex, with a unique structure that differs significantly from those of nuclear and chloroplast genomes [9].

The chloroplast genome (cpDNA), a vital plant organelle genome, holds significant value in molecular biology research. Its conserved structure and maternal inheritance facilitate phylogenetic reconstruction, species identification, and evolutionary studies. High copy numbers enable efficient genetic engineering applications, while comparative analyses reveal horizontal gene transfer events and co-evolutionary patterns with nuclear genomes. These unique characteristics make cpDNA an indispensable tool for understanding plant biodiversity, molecular breeding strategies, and fundamental cellular processes [11].

However, the absence of mitochondrial and chloroplast genome sequences in species within the *Prunus* genus presents a substantial gap in molecular biology research. Therefore, are unable to leverage mitochondrial genomes for species identification, classification, and evolutionary analysis, resulting in an incomplete understanding of the phylogenetic relationships and population history within the genus [12]. This deficiency can lead to misconceptions regarding species relationships and confusion in the taxonomic placement of the entire genus.

Recent studies have characterized the complete mitogenomes of several *Prunus* species, revealing unique features, such as gene content, organization, and variation [13]. In the cases of *P. salicina* and *P. domestica*, the sequencing and analysis of their mitogenomes have unveiled significant information regarding their genetic diversity and evolutionary adaptations. Comparative mitogenomics has highlighted the differences and similarities between these species, contributing to our understanding of their genetic makeup and providing a basis for further research into their breeding and conservation.

The origin and taxonomic classification of *P. salicina* var. *cordata* remain a subject of debate among botanists. While it is officially recognized as a variety of *P. salicina* (Japanese plum), there is considerable morphological and genetic evidence suggesting it bears a closer resemblance to *P. domestica* (European plum). Recent studies have highlighted that *P. salicina* var. *cordata* shares several morphological traits with *P. domestica*, such as a similar fruit shape and skin texture, which are distinct from those of typical *P. salicina* specimens [14]. Genetic analyses further support this observation. For example, SSR-based genetic diversity studies have shown that *P. salicina* var. *cordata* clusters more closely with *P. domestica* in certain phylogenetic trees, suggesting a closer evolutionary relationship than with *P. salicina*. Despite these similarities, *P. salicina* var. *cordata* is still classified under *P. salicina* due to the traditional taxonomic practices and some overlapping genetic markers. However, the ongoing debate is fueled by the increasing amount of genetic data that challenge this classification, urging the reconsideration of its taxonomic status. This unresolved debate underscores the urgent need for comparative organelle genome analyses to clarify the evolutionary relationships and refine the taxonomic boundaries. While *P. salicina* var. *cordata* is currently classified as a variety of *P. salicina*, its close morphological and genetic resemblance to *P. domestica* suggests a complex evolutionary history that merits further investigation. The recent literature continues to explore these relationships, potentially leading to the re-evaluation of its taxonomic position in the future.

## 2. Materials and Methods

### 2.1. Plant Materials

**Figure 1:**
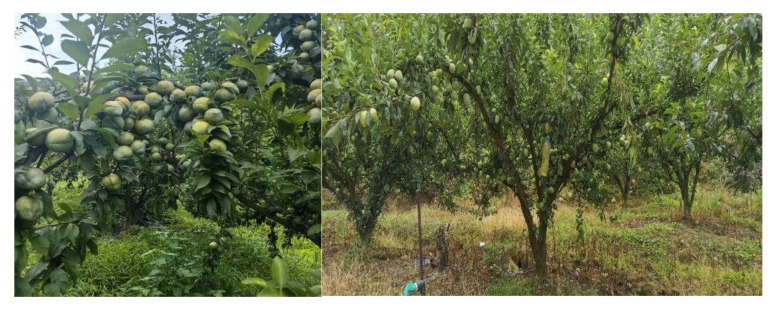
An image of the voucher specimens used in the present study taken by Liao Ru-yu.

The esteemed voucher specimens were collected from the Yongding, Fujian, China Resource Garden, under the stewardship of the distinguished Dr. LIAO Ru-yu (liaoruyu@126.com) (Figure 1). This state-of-the-art facility is situated at 24.805° N, 116.733° E. (They are a new variety of *P. salicina* var. *cordata* apple selected and bred by Liao Ruyu team of the Fruit Research Institute, Fujian Academy of Agricultural Sciences. The fruit is heart-shaped with a small cavity, greenish-yellow skin, and orange-yellow flesh. The flesh does not change brown easily and has a sweet flavor and excellent quality). The fresh leaves were gently cleansed in dihydrogen monoxide from aqueducts, and then rinsed repeatedly in sterile aqueous solution. A strict aseptic technique was maintained throughout. The leaves were rapidly dehydrated to preserve the integrity of plant metabolites. Subsequently, the specimens were placed in hermetically sealed polyethylene bags containing silica gel desiccant, providing optimal moisture control. The samples were expeditiously transferred to a laboratory grade cryogenic freezer set to −80 °C, allowing for the preservation of plant genetic material for future scientific study [15].

### 2.2. DNA Isolation and Sequencing

Genomic DNA extraction from the collected foliar samples was performed utilizing the TIANamp Genomic DNA Kit (TIANGEN Biotech, Beijing, China), adhering strictly to the manufacturer’s protocols. Approximately 200 nanograms of purified DNA was fragmented to 20,000 base pairs via adaptive focused acoustics in order to generate HiFi sequences. The DNA library was constructed and sequenced using previously reported procedures. The prepared libraries were loaded on the PacBio Sequel II sequence platform, and 1 PacBio Sequel II SMRT Cell was generated (Annoroad Gene Technology Co. Ltd., Beijing, China).

### 2.3. Mitogenome and Chloroplast Assembly and Annotation

The PacBio Sequel-II raw subread data generated from the whole genomes of *P. salicina* var. *cordata* and *P. domestica* were used for mitogenome and chloroplast assembly. High-Fidelity Circular Consensus reads (HiFi CCSs) were locally called from the subread data using SMRTLink (v 13.1). Thereafter, we refer to contigs computed by hifiasm as those computed with the whole-genome sequencing dataset, including nuclear genome assembly. We searched the mitogenome contigs with homology to the mitogenome of *Prunus* mume (NCBI reference sequence: NC_065232.1). Mitochondria and chloroplast-derived reads were extracted from the original dataset using the *P.* mume genome with minimum query coverage to the reference at 70% using minimap2 (ver. 2.28) [13]. We assembled the *P. salicina* var. *cordata* and *P. domestica* mitogenomes using Flye (ver. 2.9.4) with “pacbio-hifi” parameters. Thereafter, we used Bandage software (v0.8.1) to visualize the GFA files and manually remove the “noisy” (chloroplast or nuclear contigs) and non-target contigs, and we obtained the circular contig for *P. salicina* var. *cordata* and *P. domestica.*

The assembled mitogenome and chloroplast contig was annotated by using MITOFY [16] and tRNAscan-SE [17] with manual corrections to confirm annotation. The genome circle map was drawn by using the PMGmap online service (http://47.96.249.172:16086/home/, accessed on 10 May 2024) [18].

### 2.4. Analysis of RSCU and Repeat Sequences

We used CodonW to analyze the base composition, codon usage patterns, and relative synonymous codon usage (RSCU) in *P. salicina* var. *cordata* and *P. domestica*. The SSRs (simple sequence repeats) were assessed through MISA (https://webblast.ipk-gatersleben.de/misa/, accessed on 10 May 2024) using the following parameters: ‘1-10 2-5 3-4 4-3 5-3 6-3’. Tandemly repeated sequences were identified using Repeats Finder v4.09 software (http://tandem.bu.edu/trf/trf.submit.options.html, accessed on 10 May 2024) with default settings. Dispersed repeats were predicted employing the method in [19] (http://tandem.bu.edu/trf/trf.submit.options.html, accessed on 10 May 2024) with the parameters set to ‘Hamming Distance 3, Maximum Computed Repeats 5000, Minimal Repeats Size 30’ and filtered using an e-value cut-off of 1 × 10^−5^.

### 2.5. Phylogenetic Analyses

To conduct phylogenetic analyses, the mitogenome and chloroplast sequences’ shared genes were first extracted and concatenated using Phylosuite (v1.2.1) [20] and aligned using MAFFT (v 7.471) with default parameters [21]. The optimal model for our phylogenetic analysis was determined using ModelFinder, which employs the Akaike information criterion (AIC) to assess model fit and complexity. The model with the best balance of accuracy and simplicity based on the AIC score was selected. For maximum-likelihood phylogenetic tree construction, IQ-TREE (v2.1.2) with the GTR + F + R6 model was employed [22]. The robustness of the inferred tree topology was evaluated through 1000 ultrafast bootstraps, providing statistical support for the tree branches and allowing for the assessment of the reliability of the phylogenetic relationships. Additionally, Bayesian inference (BI) analyses were conducted using MrBayes (v3.2.6) [23]. The MCMC search included all the substitution model parameters, and two independent runs with four chains (three heated and one cold) were performed simultaneously for a minimum of 50,000,000 generations. The trees were sampled every 1000 generations until the average standard deviation of split frequencies fell below 0.01. After discarding the initial 10% trees from each MCMC run as burn-ins, the remaining trees from both the runs were combined, and a consensus tree with branch support (PP) was computed.

## 3. Results

### 3.1. Characteristics of the Mitogenomes of P. salicina var. cordata

**Figure 2:**
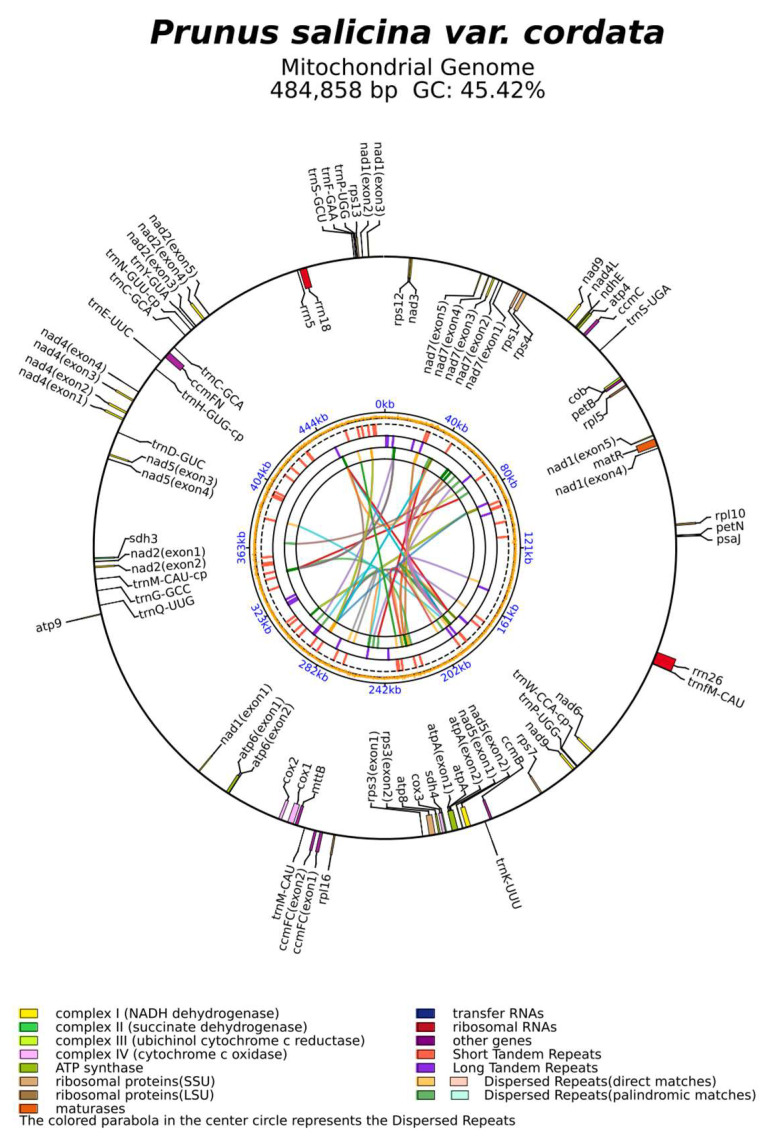
A circular map of the *P. salicina* var. *cordata* mitochondrial genomes.

The assembled migenomes of the *P. salicina* var. *cordata* and *P. domestica* mitogenomes are a single circular sequence of 484,858 bp, with an overall GC content of 45.4% (Figure 2, Table 1), which is similar to those of other *Prunus* species.

Base composition, codon usage patterns, and relative synonymous codon usage (RSCU) play a crucial role in gene expression levels and mRNA stability. They can also provide valuable insights into evolutionary patterns and phylogenetic relationships. Totals of 10,883 and 10,230 codons were found in the mitogenomes of *P. salicina* var. *cordata.*

### 3.2. The Amino Acids of P. salicina var. cordata

**Figure 3:**
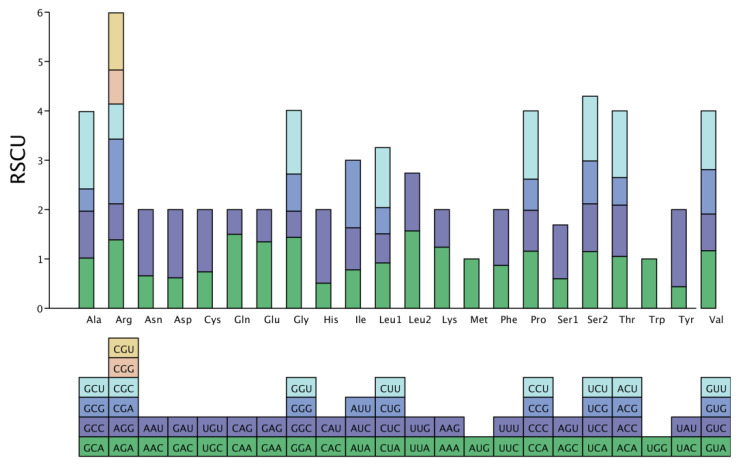
A bar plot showing the distribution of codon content for the amino acids of *P. salicina* var. *cordata*.

Interestingly, certain amino acids show higher usage rates within these codons. In *P. salicina* var. *cordata*, Isoleucine, Glycine, and Arginine are the most common amino acids, making up 7.89%, 6.73%, and 6.68% of the codons, respectively. Cysteine is the least common, at just 1.42% (Figure 3). Similarly, in *P. domestica*, Isoleucine, Glycine, and Arginine are the most prevalent, comprising 7.88%, 6.79%, and 6.72% of the codons, respectively, while Cysteine is the least prevalent at 1.46% [24]. These findings emphasize the importance of codon usage in deciphering evolutionary patterns and gaining insights into phylogenetic relationships. The different usage rates of amino acids in codons can be influenced by a variety of factors, including the gene’s function, the organism’s evolutionary history, and the environmental conditions. By understanding these factors, we can better understand the mechanisms that drive evolution and the relationships between different species.

### 3.3. Phylogenetic Analysis

**Figure 4:**
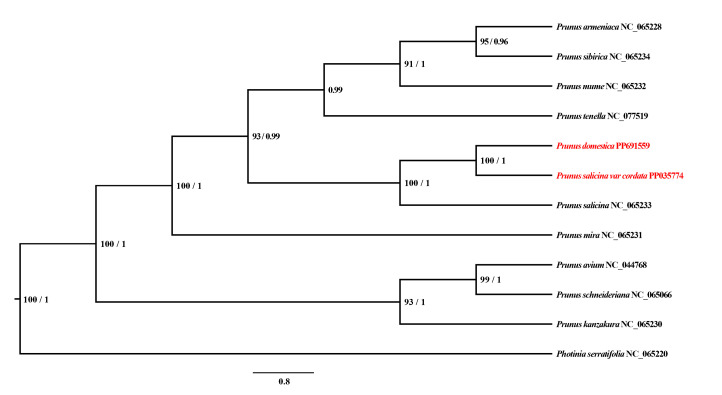
A reconstruction of a phylogenetic tree determined via maximum likelihood and Bayesian inference methods based on the PCGs of 12 mitogenomes. *P. salicina* in this study is marked in red font.

The phylogenetic tree presented elucidates the evolutionary relationships within the *Prunus* genus, emphasizing the genetic connections among various species, including *P. domestica* and its close relatives. The high bootstrap values across the tree indicate strong support for these inferred phylogenetic relationships, suggesting the robustness and reliability of this analysis. *P. domestica* appears closely related to *P. salicina* var. *cordata* and *P. salicina*. (Figure 4)

### 3.4. Characteristics of the Chloroplast of P. salicina var. cordata

**Figure 5:**
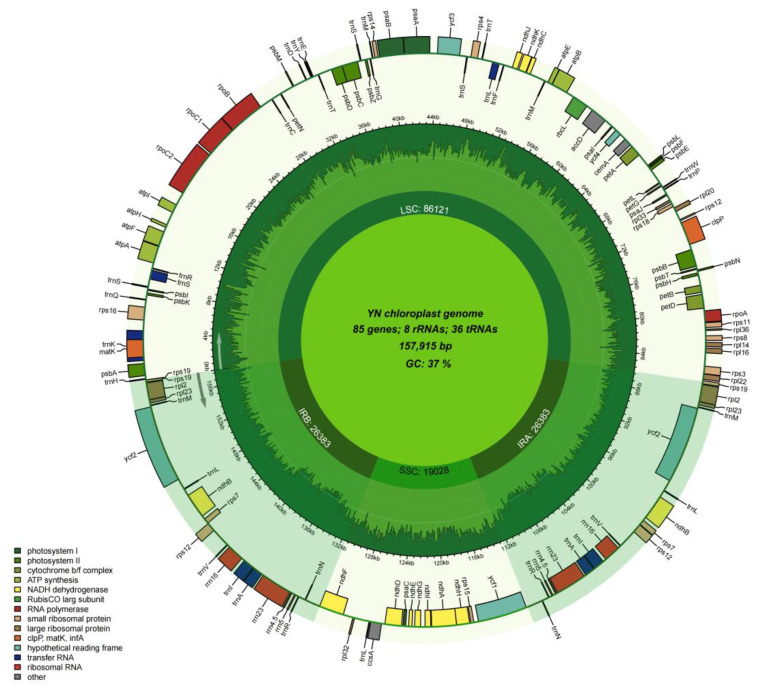
A circular map of the *P. salicina* var. *cordata* Chloroplastare genomes.

The chloroplast genome of *P. salicina* var. *cordata* was assembled and annotated, revealing a typical quadripartite structure consisting of a large single-copy (LSC) region (86,121 bp), a small single-copy (SSC) region (19,028 bp), and two inverted repeat (IR) regions (26,383 bp each), with a total length of 157,915 bp (Figure 5). The genome encodes 132 functional genes, including 85 protein-coding genes, 36 tRNA genes, and 8 rRNA genes. The overall GC content was 37%, with variations observed across the regions (LSC: 34.5%; SSC: 29.1%; and IR: 42.9%).

Comparative analysis identified 51 long repetitive sequences (≥30 bp) and 81 simple sequence repeats (SSRs) predominantly located in non-coding regions. Phylogenetic reconstruction based on 78 shared protein-coding genes placed *P. salicina* var. *cordata* in a clade with *P. salicina* var. *salicina*, supported by high bootstrap values (100%). Notably, structural collinearity and synonymous substitution rate (Ks) analyses indicated conserved synteny with other *Prunus* species, though minor rearrangements were detected in the LSC/IR junctions. These results suggest limited plastid divergence between the *P. salicina* varieties, possibly influenced by historical hybridization or incomplete lineage sorting.

### 3.5. Phylogenetic Tree Analysis of P. salicina var. cordata Chloroplast Genome

**Figure 6:**
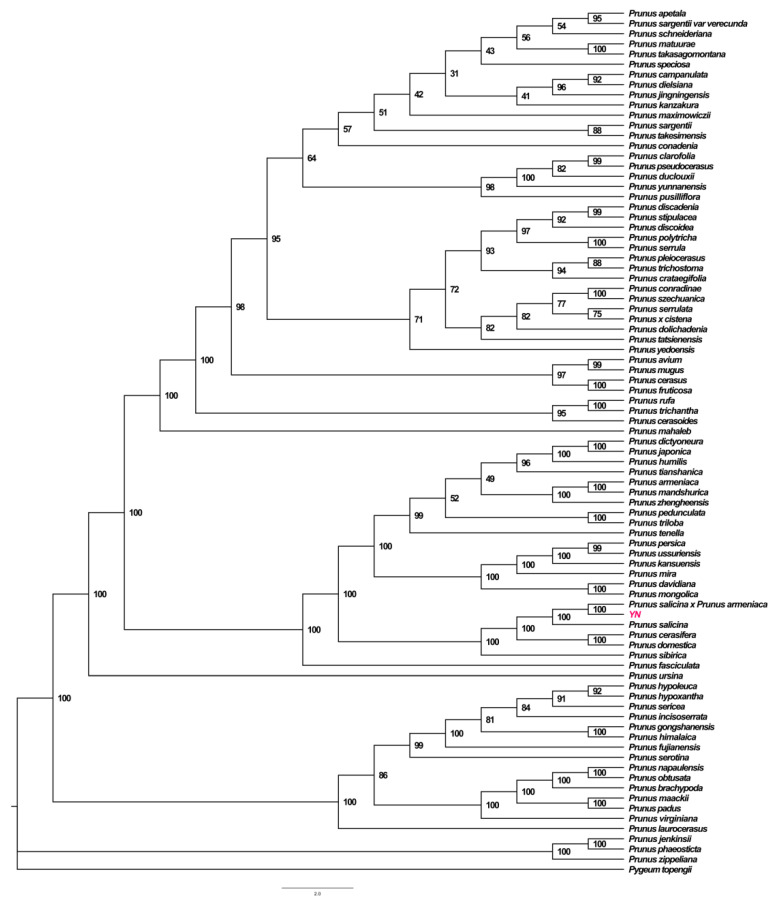
The reconstruction of a phylogenetic tree determined via maximum likelihood and Bayesian inference methods based on PCGs of 100 Chloroplastare. *P. salicina* var. *cordata* in this study is marked in red font.

Phylogenetic reconstruction using the maximum likelihood (ML) and Bayesian inference (BI) methods based on 78 conserved protein-coding genes from 100 species across *Prunus* and related genera robustly resolved the evolutionary placement of *P. salicina* var. *cordata* (Figure 6). The ML tree (lnL = −235,678) shows strong nodal support (bootstrap ≥ 95%) for a monophyletic clade containing *P. salicina* var. *cordata, P. salicina* var. *salicina*, and *P. domestica*, with *P. cerasifera* as their closest sister. The Bayesian posterior probabilities (PPs = 1.0) corroborated this topology, confirming minimal plastid divergence between the *P. salicina* varieties.

Notably, *P. salicina* var. *cordata* formed a distinct subclade with *P. salicina* × *Prunus armeniaca* (100% bootstrap), sharing a recent common ancestor (estimated divergence time: ~1.2 Mya). Collinearity analysis (Figure 6) revealed a highly conserved gene order across the *P. salicina* complex, except for minor inversions near the ndhF-ccsA junction. The synonymous substitution rates (Ks) between the *P. salicina* varieties averaged 0.003, significantly lower than interspecific comparisons (Ks > 0.05), supporting ongoing plastid introgression or incomplete reproductive isolation. These results align with the chloroplast capture scenarios, potentially driven by historical hybridization events within the East Asian Prunus populations.

## 4. Discussion

The mitochondrial genomes (mitogenomes) of *P. salicina* var. *cordata* were assembled as single circular sequences of 484,858 bp, with identical GC contents (45.4%) and near-identical AT contents (54.6%). These values align closely with those of other *Prunus* species (e.g., *P. avium*: GC 45.7%; *P. mume*: GC 45.5%), suggesting conserved genomic architecture across the genus [15]. The structural and compositional consistency, particularly in GC/AT ratios, implies strong evolutionary constraints on the mitogenome organization in Prunus, likely driven by functional necessities such as energy metabolism or replication efficiency. For instance, mitochondrial intermediary metabolism is central to ATP synthesis and Fe–S cluster biosynthesis, a process conserved across eukaryotes. Notably, the mitogenome length of *P. salicina* var. *cordata* and *P. domestica* falls within the observed range for Prunus (389,709–535,727 bp), with variations potentially attributed to lineage-specific insertions, deletions, or horizontal gene transfer events [25]. These findings reinforce the utility of mitogenomes in delineating intra-generic relationships, while highlighting the need for functional studies to elucidate mechanisms underlying structural stability.

The codon usage patterns in the *P. salicina* var. *cordata* mitogenomes revealed a pronounced bias toward specific amino acids, notably Isoleucine, Glycine, and Arginine, while Cysteine remained the least frequent. This consistency across both the taxa suggests shared evolutionary pressures, possibly linked to translational efficiency or tRNA abundance. The high usage of hydrophobic residues (e.g., Isoleucine) may reflect adaptations to mitochondrial membrane protein synthesis, whereas the low Cysteine content could relate to redox stress mitigation in organellar environments. Such codon bias patterns are likely influenced by mutation–selection equilibrium, with synonymous substitutions constrained by functional requirements; in addition, this bias likely optimizes mitochondrial membrane protein synthesis, as hydrophobic residues dominate the ETC complexes (NADH dehydrogenase), ensuring proper membrane embedding and proton channeling [26,27]. These results underscore the role of mitogenome codon usage in shaping evolutionary trajectories, offering a framework for comparative studies on adaptive divergence within Prunus.

*P. domestica*, commonly known as the European plum, shares a particularly close genetic relationship with *P. salicina* var. *cordata*, a variety of Japanese plum. The strong bootstrap support indicates that these two species have a recent common ancestor. A recent study [28] has provided insights into the genetic closeness of these species through chloroplast and nuclear DNA analyses. These studies found that *P. domestica* and *P. salicina* var. *cordata* exhibit high levels of genetic similarity, likely due to historical hybridization events and shared evolutionary pressures. Physiologically, such hybridization may introduce alleles enhancing stress tolerance; for instance, *P. domestica*’s cold hardiness genes could have introgressed into *P. salicina* var. *cordata*, aiding its adaptation to temperate climates [29]. *P. salicina*, another variety of Japanese plum, also shows a close relationship with *P. domestica*. This relationship is supported by both the morphological and genetic data. According to other report, SSR marker analyses have demonstrated significant genetic overlap between *P. domestica* and *P. salicina* [30]. This genetic overlap suggests that these species may have undergone extensive gene flow, possibly facilitated by human cultivation practices that have historically brought these species into contact.

The genetic closeness between *P. domestica*, *P. salicina* var. *cordata*, and *P. salicina* has important implications for our understanding of their evolutionary history and domestication. The high genetic similarity suggests that these species have likely exchanged genetic material through hybridization. This hybridization is supported by the work of other researchers, who found evidence of interspecific gene flow among *Prunus* species using genome-wide SNP data [31].

Furthermore, the close genetic relationships observed may have been influenced by their domestication and cultivation. Domesticated *Prunus* species often exhibit reduced genetic diversity compared to their wild relatives, as a result of selective breeding for desirable traits. This phenomenon has been documented by other studies, which highlighted the impact of domestication on the genetic diversity of *Prunus* species [32].

The geographical distribution of these species also plays a role in their genetic relationships. *P. domestica* is primarily cultivated in Europe, while *P. salicina* and its variety *P. salicina* var cordata are more common in East Asia. The historical movement of these species along trade routes and through human migration has likely facilitated gene flow, contributing to their genetic similarity. Previously research discussed how ancient trade routes enabled the spread and hybridization of *Prunus* species, further supporting the close genetic relationships observed in phylogenetic analysis [33].

The chloroplast genomes of *P. salicina* var. *cordata* exhibited typical quadripartite structures, with a conserved gene content and a GC content comparable to that of other *Prunus* species. Structural collinearity and shared SSR/repeat distributions across the taxa suggest strong evolutionary conservation, yet minor inversions near the ndhF-ccsA junctions hint at lineage-specific structural plasticity. Phylogenetic reconstruction based on 100 species placed *P. salicina* var. *cordata* within a monophyletic clade alongside *P. domestica* and *P. salicina* var. salicina, with a divergence time estimated at ~1.2 Mya. The exceptionally low Ks values between the varieties and strong bootstrap support for their sister relationship strongly imply recent plastid introgression, likely mediated by historical hybridization in the East Asian Prunus populations [34]. These findings align with the chloroplast capture scenarios, where cytoplasmic genomes are transferred across species boundaries without nuclear gene flow, offering a plausible explanation for the discordance between the organellar and nuclear phylogenies observed in some Rosaceae lineages.

The parallel conservation of the mitochondrial and chloroplast genomes in *P. salicina* var. *cordata* and *P. domestica* highlights the contrasting evolutionary dynamics of cytoplasmic genomes. While mitogenomes exhibit structural and compositional stasis, chloroplasts show localized rearrangements and evidence of historical hybridization [35]. This dichotomy may reflect differences in the mutation rates, the repair mechanisms, and the selective pressures acting on the two organelles. The congruence in phylogenetic placement across both the genomes nevertheless reinforces their utility in resolving shallow evolutionary splits, particularly in recently diverged taxa. Future studies should integrate the nuclear genomic data to disentangle the roles of hybridization, selection, and demography in shaping the complex evolutionary history of Prunus. Such efforts will clarify whether the observed paleoenvironments patterns reflect neutral processes or adaptive responses to ecological pressures in East Asia’s dynamic paleoenvironments.

## 5. Conclusions

This study deciphers the first mitochondrial (484,858 bp) and chloroplast (157,915 bp) genomes of *P. salicina* var. cordata, revealing conserved structures and a GC content (37%) akin to those of related species. Phylogenetic analysis confirmed close kinship between *P. domestica* and *P. salicina*, with inferred historical gene flow, advancing genomic resources for resolving Prunus phylogeny and evolutionary dynamics.

## Figures and Tables

**Table 1:** The reference mitogenome sequences that were used to align the sequence in this study.

Family	Genus	Organism	Length	AT%	GC%	ID
*Gunneridae*	*Prunus*	Prunus avium	389,709	54.3	45.7	NC_044768.1
		Prunus kanzakura	422,215	54.4	45.6	NC_065230.1
		Prunus mira	429,732	54.4	45.6	NC_065231.1
		Prunus schneideriana	434,334	54.3	45.7	NC_065066.1
		Prunus tenella	452,158	54.4	45.6	NC_077519.1
		*P. domestica*	484,858	54.6	45.4	PP691559
		*P. salicina* var. *cordata*	484,858	54.6	45.4	PP035774.1
		*P. salicina*	508,005	54.5	45.5	NC_065233.1
		Prunus sibirica	510,187	54.6	45.4	NC_065234.1
		Prunus armeniaca	510,346	54.5	45.5	NC_065228.1
		Prunus mume	535,727	54.5	45.5	NC_065232.1
	*Photinia*	*Photinia serratifolia*	473,561	54.8	45.2	NC_065220.1

## Data Availability

The genome sequence data that support the findings of this study are openly available in GenBank of the NCBI (https://www.ncbi.nlm.nih.gov/) under accession no. PP035774 (accessed on 15 May 2024).
